# Rebuilding life after migration: Research protocol of a mixed methods study on settlement experiences of refugee and migrant youth

**DOI:** 10.1371/journal.pone.0285023

**Published:** 2023-04-28

**Authors:** Tahereh Ziaian, Teresa Puvimanasinghe, Emily Miller, Martha Augoustinos, Adrian Esterman, Michelle Baddeley, Nancy Arthur, Helena de Anstiss, Eugenia Tsoulis, Tamara Stewart-Jones, Effat Ghassemi, Tara Pir

**Affiliations:** 1 University of South Australia, Justice and Society, Adelaide, Australia; 2 Centre for Workplace Excellence, University of South Australia, Adelaide, Australia; 3 University of South Australia, Allied Health & Human Performance, Adelaide, Australia; 4 University of Technology Sydney, Ultimo, Australia; 5 University of South Australia, Business, Adelaide, Australia; 6 Multicultural Youth South Australia, Adelaide, South Australia, Australia; 7 Australian Migrant Resource Centre, Adelaide, South Australia, Australia; 8 Newcomer Centre of Peel, Mississauga, Ontario, Canada; 9 Institute for Multicultural Counseling and Education Services, Los Angeles, California, United States of America; University of Greenwich, UNITED KINGDOM

## Abstract

Internationally, there is an urgent need to understand factors promoting successful settlement and integration of people with forced or voluntary migration experiences (i.e., refugee and non-refugee migrants). This paper provides a protocol of a mixed-methods investigation of contextual factors of successful settlement and service utilization of youth and their families, as young people could be at higher risk due to stressors associated with pre-migration trauma, post-migration settlement stressors, and adolescent development. This large-scale mixed-methods study will be conducted across three countries. A questionnaire survey will seek responses from 1200 youth aged 15–24 years residing in South Australia, Ontario, Canada, and California, United States of America. The qualitative component of the study will comprise 54 focus groups (324 participants) with youth and their parents/caregivers. The study design allows a range of important phenomena (e.g., different migration pathways and settlement countries) and key questions (e.g., regarding the intersection of migration, settlement, and wellbeing) to be addressed. It also allows for generalizability of findings to be tested across different communities and countries. Findings will support recommendations for policy and practice and may be generalized to advance research with youth and their families. This study is one of the largest, most comprehensive studies of youth settlement.

## Introduction

In Australia and internationally there is a pressing need to understand the factors that promote successful settlement of young people and their families after migration. A socially welcoming, inclusive, and productive society strives to support all members and enables equitable participation in social institutions such as education and employment. However, various contributing factors can impact settlement negatively, including at the broadest level in policy and law that restrict access to opportunity, at the society level with negative attitudes and discrimination from the broader settlement society, and at the personal level where negative experiences may lead migrants to develop mistrust in systems and professionals working within them [1]. These challenges are in addition to negative experiences pre-migration, recovery and healing from which can be hampered by post-migration experiences, particularly important for those with refugee backgrounds [2]. Ongoing separation from loved ones due to migration regulations and laws is another compounding factor [3,4]. Migration continues to be a dynamic but central element of societies around the globe, and research to investigate these issues and provide evidence to support positive practices and policy in settlement countries is therefore needed. Young people may especially be at higher risk as pre- and post-migration stressors impact family relationships, identity development, psychological health, and wellbeing [5,6].

Evidence suggests that youth from migrant backgrounds have a higher risk for psychological distress, associated with settlement stressors, changes in family relationships, and cultural change and adaptation [7], issues which are even more pertinent for migrant youth with refugee backgrounds [8]. Barriers to accessing help for these challenges include cultural stigma, differing understandings of mental health, and a lack of knowledge about available services [9]. Adolescents have been shown to experience common barriers to seeking help for mental health issues, however, a majority of programs of support are facilitated through education settings [10] and may not provide culturally informed and responsive practices that work for young people from culturally and linguistically diverse migrant and refugee backgrounds [11]. Further barriers can include a distrust of services in general and confidentiality concerns [12]. Therefore, it is crucial that policies governing practice, and services providing support for youth settlement, are informed by research that identifies and provides evidence of what works in different contexts and with different populations.

Further research is required to determine what factors are persistent across migrant populations and therefore which policies and practices have wider applicability across settlement contexts. This study will investigate a range of contributing factors with a particular focus on how support services promote psychological health and wellbeing of youth settling into Australia, Canada, and the United States of America (USA), with a view to influencing settlement and multicultural mental health policy and practice. These services may include multicultural services providing specialized settlement supports, and/or other services that young people and families may come into contact with during settlement, such as education, health, or employment services.

The proposed research will provide a unique opportunity to investigate the settlement experiences and wellbeing of Australian youth and families, alongside those in Canada and the USA. These three different contexts provide environments where there is relatively high immigration, and therefore the impact of local policies and practices can be considered within the broader context of global migration. The research brings together a team of internationally recognized experts in immigration, settlement, acculturation, multicultural mental health, multicultural service delivery, behavioral and cultural economy, and epidemiology, with key industry partners from the multicultural services sector in the three countries.

### International migration: Global and local trends

Global migration and mobility has increased in terms of numbers alongside global population growth and in 2020 there were around 281 million international migrants around the world [13]. Growth in the numbers of forcibly displaced people, including refugees, has also seen a steep rise over the last few years and currently stands at an estimated 103 million people [14]. Therefore, it is critical that research is conducted to understand experiences of migration and settlement, and for research to provide evidence to inform policy and practice.

The United Nations Refugee Convention defines a “refugee” as: “a person who is outside his/her country of nationality or habitual residence, has a well-founded fear of persecution because of his/her race, religion, nationality, membership of a particular social group or political opinion, and is unable or unwilling to avail him or herself of the protection of that country, or to return there, for fear of persecution” [15]. The Convention distinguishes people who are forced to leave their countries from other migrants who leave their countries voluntarily. Though these two groups share many commonalities, such as in identity development resulting from moving to foreign cultures with attendant losses, people forced to leave their countries have often endured complex trauma and stress associated with war, persecution, and forced exile. This study therefore investigates the experiences of migrants with and without refugee backgrounds in order to understand the common and contrasting experiences across these two groups.

Although the study will identify any similarities and differences of experience between these two groups, it is also important to note that neither one is a homogenous group, and neither are they mutually exclusive. Migration regulations, policies and laws around the globe affect how and when people can cross borders, and under what circumstances, as well as what rights and responsibilities they have in settlement. Although refugee resettlement does require that people have been legally afforded the categorization of refugee [16], there are many people with refugee-like experiences who migrate via other mechanisms. Individuals may develop a sense of identity that aligns with their migration pathway, or they may not [17]. In addition, the push and pull factors, and the life experiences in different locations around the world, are highly heterogenous. Someone who may be categorized as refugee or as migrant may have vastly different experiences to another person who has been similarly categorized [18] and a dichotomous approach to migration research can obscure some of these details whilst inadvertently reinforcing negative implications [19]. This study aims to identify the intersections of migration experiences across these categories, and to understand pertinent contextual and experiential factors are affecting settlement.

The USA is home to the highest number of immigrants of any nation globally, accounting for around 15% of the total population [20]. In Australia, although the overall number is smaller, the proportion of the population who migrated to Australia is double that of the USA at nearly 30% [21]. Almost half of all Australians are either born overseas or have at least one parent born overseas [22]. Canada is a nation with rising migrant populations, with latest figures showing it has the highest immigration rates among the G7, making up around 23% of the total population; it is predicted that in the coming years Canada will have a proportionally similar migrant population to that of Australia today [23]. All three countries not only have relatively high immigration, but also contribute to refugee resettlement in consultation with the United Nations [24].

As further described in the below sections, this study will draw on data with migrant and refugee populations in these three countries. The relatively high immigration rates, and therefore the composition of the societies in these locations will enable assessment of the various factors that impact settlement within different local contexts whilst also considering shared experiences of people migrating from particular regions. The local policies and practices in these three countries, as well as the historical and contemporary contexts will be crucial elements of the study, including: issues such as racism or social in/exclusion; regulations relating to and understandings of migrants and refugees; and, barriers to integration in relation to education and employment opportunities [18,25,26].

### Settlement challenges for youth and families

Much of the literature on migration and its impact on mental health has focused on trauma-related psychopathology, neglecting the broader psychological, familial, social, and cultural dimensions of the settlement experience [27,28]. However, during the last decade there has been growth in research investigating the socio-cultural factors that provide support and protection for young people in settlement [29], with growing recognition of the intersection of social support and other social determinants of health during settlement [30–33]. For young people, experiences within the broader society, at school or in social settings, combine with experiences within the family to impact mental health and wellbeing [34–37].

Parents face unique challenges in settlement including dramatic changes in family structure, dynamics, and roles, often in a context of social isolation, limited family, peer, and community support systems, and high societal expectation to adjust quickly, within a broader context of transnational separation and belonging [27,38–40]. One of the most salient issues for many families is intergenerational conflict during the acculturation process [12,41] attributed to varying degrees of change and proficiency of family members in navigating a new cultural context. Differences in cultural adaptations can result in intergenerational conflict and significant family problems [5,42], as a result of what has been termed the “acculturation gap”—identified as one of the most persistent issues facing families [41]. This can be made harder when they have limited English language skills and are dependent on their children to act as language brokers for the family [38].

In addition to challenges within families, multiple risks for the wellbeing of youth with settlement experiences have been attributed to migration and settlement processes, including limited majority-language skills (English or French in the case of the countries in this study), difficulty negotiating a new culture and systems, pre-migration experiences of trauma, loss of familiar support systems and networks, grief and loss, identity confusion, experiences of discrimination, school adjustment problems, and low social participation [43–47]. A range of protective factors have been identified including through family and community connection and support, and through a sense of belonging at school [48], and cross-cultural adaptations of both young people and their families is central [49].

Youth need access to strong support systems–social and community support systems as well as health, educational and employment support systems–as they negotiate the challenges associated with settlement and cultural transition, as they face the developmental issues common to all youth, and as they manage the concerns unique to them [44,46]. Available research suggests that most of these youth do not access mental health and support services [12,43,50–53], and that available support programs may not meet their needs [54]. This research will explore the nature and scope of service utilization by migrant and refugee youth and their families for a wide range of psychosocial, familial, cultural problems, thereby providing a more comprehensive knowledge base from which to improve service provision.

### Theoretical and methodological approach

The term “integration” has taken on a range of definitions in the literature, in popular and media culture, and in policy rhetoric, and therefore it is crucial that we identify our understanding and use of the term [55]. Integration has typically been understood as a mode of incorporation of migrants into settlement societies, taking into account linguistic, cultural, and social aspects as well as more practical matters such as employment, education, and health outcomes [56]. Often migrants themselves are a focus for research investigating integration outcomes, and data are used to attempt to measure the rate of integration in terms of various measures such as income, health, or education levels [40]. However, several scholars have noted that the actions of a settlement society towards newcomers are central to integration [57,58]. It is a combination of inclusive and welcoming societies as well as local policies that impact newcomers’ experiences of settlement [26,59].

Integration as a term, therefore, can be used broadly to indicate migration, settlement, and cross-cultural experiences of individuals, groups, and societies. It can both encompass and be compared to “assimilation”, a term used to describe when newcomers adapt to a new society and environment specifically by conforming and taking on the norms and beliefs of that context and forgoing heritage practices [60]. In some cases, as dictated by the localized context, these terms may be taken to be almost the same; outcomes or measures of integration often require that migrants have access to opportunity for education, employment, housing, or social connection that may require assimilation to majority norms if the receiving society is not an inclusive environment for linguistic or cultural diversity [61].

In this study, we use the term integration to indicate broad settlement experiences of migrants as they settle into a new society, situated within the social and historical context of the society they settle into. We incorporate an understanding of integration that considers cross-cultural adaptations and acculturation of both those settling as well as attitudes and behaviors of members of the receiving society [60], alongside other measures or markers of integration such as social connections, education or employment outcomes and aspirations [56]. We investigate the complex interplay of cultural and political factors that affect experiences of integration. The research also recognizes the diversity of the settlement experience by drawing on the Segmented Assimilation Model [25,62] to assess youth integration in Australia, Canada, and the USA, acknowledging that some youths are experiencing structural barriers which limit opportunities, while others experience upward mobility.

In the Segmented Assimilation Model, the emphasis is on the contextual factors within a settlement society that impact how migrants and their children can access opportunity and upward mobility (or not) [25]. This approach emphasizes contextual, structural, and cultural factors that affect integration [25,62]. Here, ‘successful’ integration is taken to mean migrants’ maintenance of heritage norms and beliefs alongside acceptance and adaptations (otherwise termed acculturation) to the majority norms and beliefs in a settlement country [60]. The research focuses both on the personal attitudes and adaptations of young people and their families as well as the contextual and experiential factors that impact them, and therefore the focus for integration is both on those migrating and settling as well as on the receiving society.

We argue that settlement trajectories and outcomes are influenced by the extent to which youth experience opportunities to achieve their goals, versus structural barriers that limit these opportunities. In accord with the Segmented Assimilation Model, the first aim of the research is to identify contextual factors that influence settlement and wellbeing [25,62], focusing on three domains of context that differ across groups and individuals: (1) experience, knowledge, strengths, and aspirations, (2) context in the host country, and (3) responsiveness of the host community to the individual and group [62]. We expect the integration pathways to be affected by these contextual factors but seek to better understand the perspectives of youth and their families.

The research also draws on Advocacy/Transformative methodology [63], which provides guidance for researchers working in culturally complex communities, to improve unsatisfactory social conditions and outcomes for socially marginalized population groups [64]. This approach importantly places priority on the lived experience of individuals and groups and adopts a transformative lens that sees action as an important outcome of research [63,64]. As such, this research project seeks to improve conditions for youth and their families via the researchers’ advocacy activities with the settlement communities, as well as with policymakers, service planners, and professionals who deliver direct services to forced and voluntary migrants.

### The study

Improved understanding of settlement challenges is critical to young people’s longer-term settlement, and that of their families, into Australian, Canadian, and American societies in this study, as well as other settlement countries globally. This research project addresses this need and will be the first of its kind undertaken in Australia and one of the largest and most comprehensive studies of youth settlement in the world. It focuses on similarities and differences in experience for migrants and refugees across the three countries in terms of immigration and settlement policies and supports, differing socio-cultural environments, and national histories. The research will identify whether there are persisting challenges or processes of support that work for young people and families in settlement across these contexts. The research will also identify important contemporary challenges and coping mechanisms, such as may have occurred throughout COVID-19 times.

The findings will be translated into a set of policy recommendations to contribute to successful settlement, social and economic participation and psychological health and wellbeing of youth and families with refugee and migrant backgrounds. Key stakeholders, particularly policymakers and service planners, and settlement communities themselves will be provided with evidence to develop policy and practice guidelines to improve service development and provision of intervention strategies.

### Aims

We aim to conduct a mixed-method study comprising a large-scale quantitative survey study and a qualitative Focus Group (FG) study to examine settlement experiences, psychological health and wellbeing, and the role of support services in fostering the positive integration and settlement of youth in all three countries. The results will provide new knowledge and better understanding of support services within an international context.

The proposed research will address the following four aims:

1. To conduct an in-depth investigation of the experiences of youth and the diverse contexts of migration to inform knowledge and evidence to improve their settlement and psychological wellbeing.
2. To identify contextual factors that youth and their families identify as relevant for settlement and psychological wellbeing of youth, and to examine the relationships between successful settlement and wellbeing.
3. To investigate the nature, scope and effectiveness of support services currently accessed by youth and their families.
4. To provide recommendations for effective research communication and dissemination strategies to impact settlement communities, policymakers and service providers across the mental health and social services sector, and position future research in the field of settlement.

### Methods

#### Selection criteria

**Fig 1:**
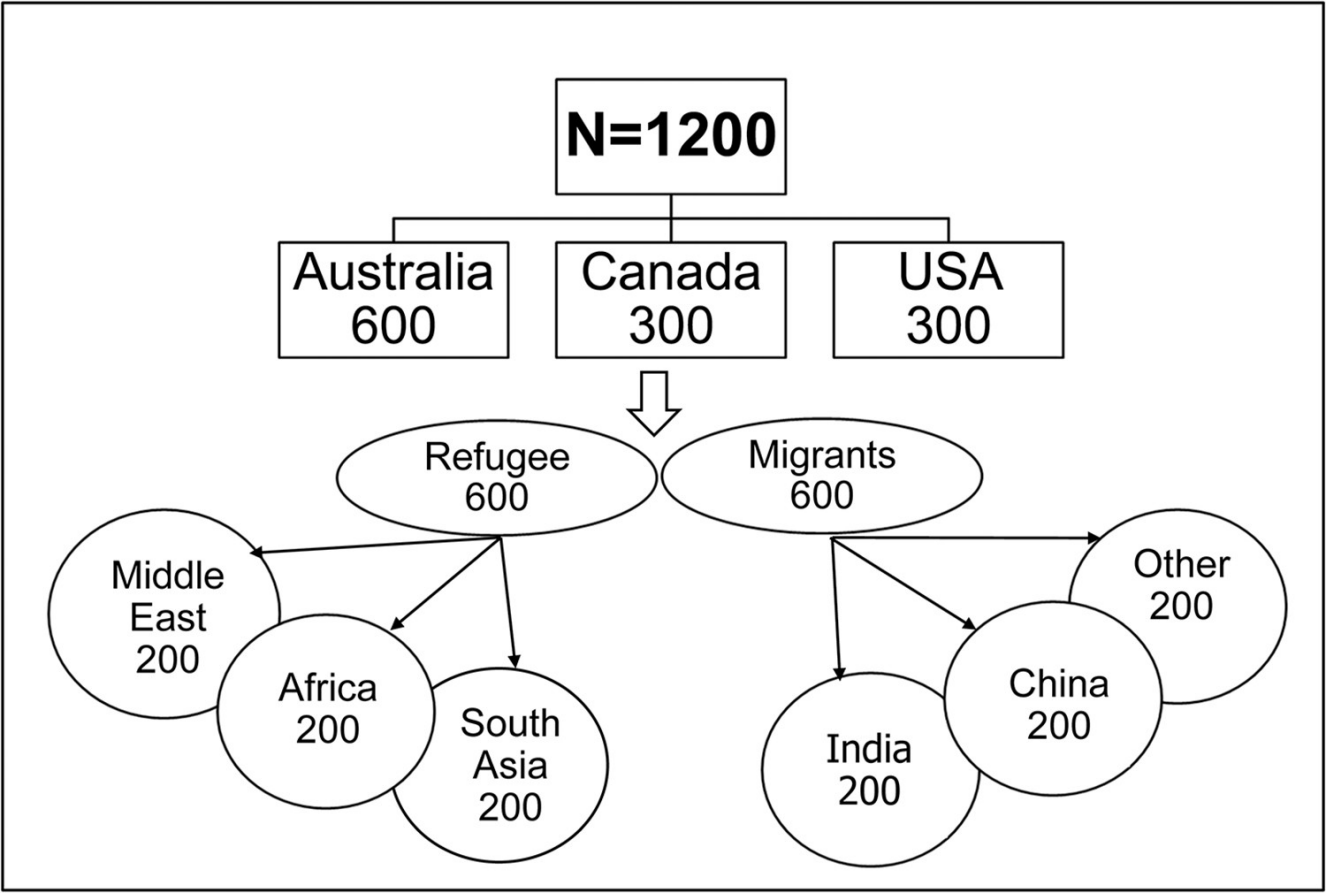
Design of the survey study.

A survey will be conducted with 1200 youth aged 15–24 years. While 600 of these youth participants will be residents of South Australia, 300 will reside in Canada (Province of Ontario) and 300 in the USA (State of California) (Fig 1). Of the 1200 youth participants, we will seek even representation of youth from refugee (n = 600) and non-refugee (n = 600) migrant backgrounds, whilst noting, as discussed above, that there can be intersections of refugee and migrant experiences. The participants will be drawn from across a large number of refugee and migrant communities with the aim of including representation from among the major countries for immigration during this selected timeframe, such as countries in Africa, the Middle East, Latin America and the Caribbean, and Southern and Eastern Asia [23,38,65].

**Fig 2:**
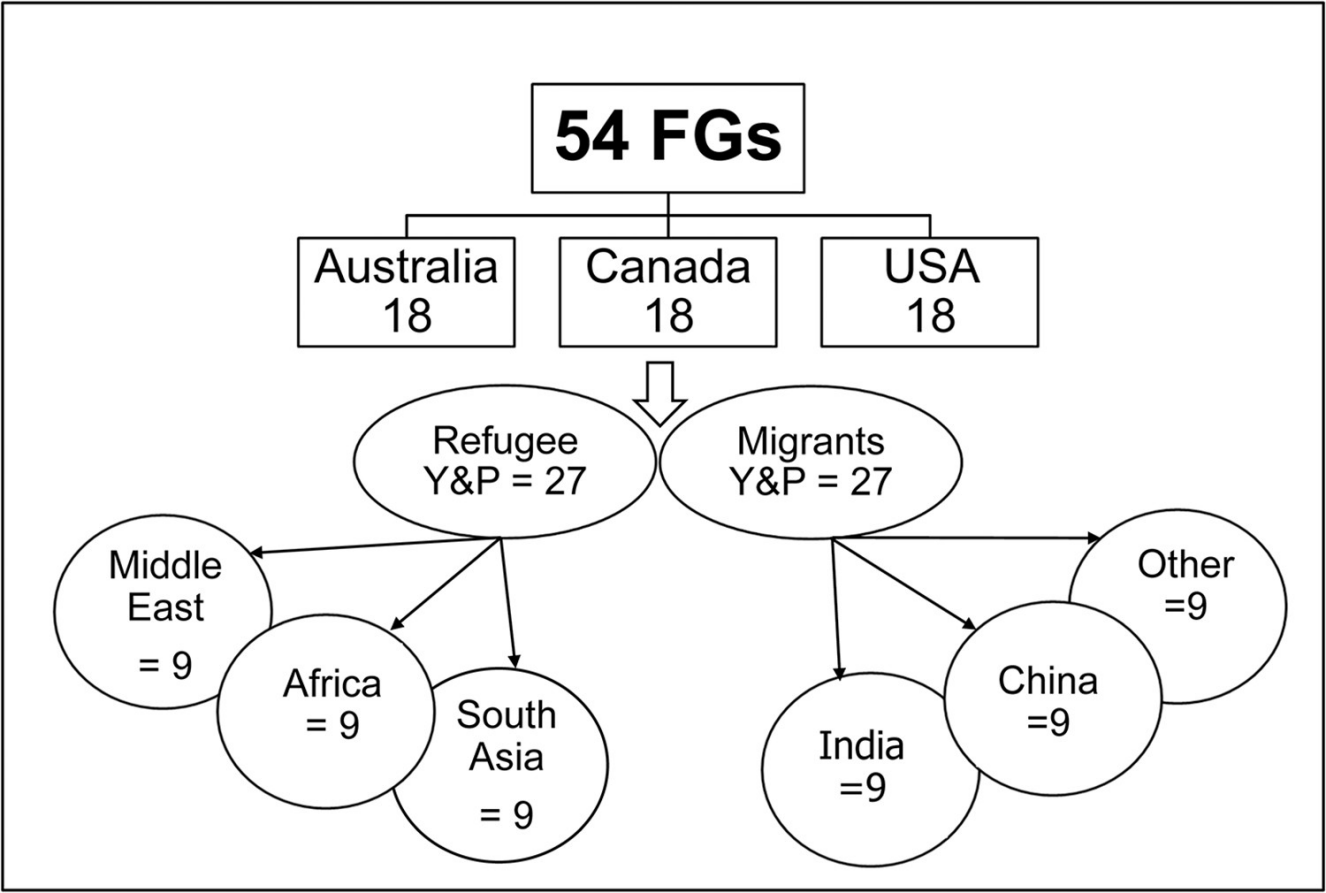
Design of the focus group (FG) study.

The FG study will comprise 54 focus groups conducted in the three project sites (18 FGs per site, Fig 2). In each project site, FGs will be conducted with parents and youth separately. Youth FGs will be conducted in English, and organized separately for females and males. FGs with parents will also be gender-specific, taking into consideration the migration context of participants (whether immigration was forced or by choice). Considering parents’ preference to speak in their heritage language, accredited interpreters will be offered to all parent participants. Special consideration will be given to the cultural backgrounds and language preferences of FG participants to ensure that not more than three languages are spoken at each parent FG, to facilitate lively discussion. With a minimum of five participants per group and a maximum of seven, between 23 and 32 percent of the entire survey sample will be represented in the FG study. With an average of six participants per group, we expect around 324 participants in the FG study (108 per site).

In both survey and FG components of the study, we will select youth and parents who have immigrated to Australia, Canada, or the USA in the past 1–15 years because prior research indicates that the initial and middle stages of migration and resettlement present unique settlement challenges and strengths compared to the later years [66]. We will exclude those with less than 12 months in their settlement country because the stressors during the first year of settlement would render them less suitable for the research study [64].

The majority of youth participants will be first generation. However, the criterion for inclusion regarding time in country is up to 15 years in the settlement country, and the minimum age for participation is 15, therefore there may be some youth who were born at the point of arrival in the settlement country and hence technically second generation. Children or adolescents who migrate are sometimes also referred to as 1.5 generation as they have some formative experiences in a previous country and some in the settlement country.

Participants will be permanent residents or citizens of Australia, Canada, or the USA, at the time of participation. Those having any kind of temporary migration status would be excluded from the study because their status in the host country is uncertain and some of their main challenges are likely to arise from the uncertainty of their migration status. Temporary residents continue to be ineligible for many services afforded to others as well as the distress associated with uncertain migration status render those with temporary visas less suitable for the study [67–69]. While we firmly believe that the issue of temporary migration (i.e., asylum seekers and temporary workers) is a worthy study, such is outside the scope of this research study.

The majority of youth would be expected to engage in some sort of formal study, (e.g., secondary school, college, university), however, youth who are not engaged in study are also included. Families with more than one child will have the option of having up to two of their children participate. The selection method will be the “birthday technique” i.e., two children with birthdays closest to the interview date being included. The criteria for ethnicity will be self-ascribed ethnic/cultural/national origin.

#### Sampling method

In this research study, probability sampling methods are not possible due to a sampling frame not being available. Instead, we will combine convenience and snowball sampling to recruit participants. These methods are generally used when the desired characteristics of the sample are uncommon, when the target population is difficult to access, and when non-probability sampling methods are not possible, all of which apply to settlement populations [63]. To enhance representativeness of study samples, we will request key stakeholders with extensive contact with these populations to assist with recruiting initial participants who in turn will refer other potential participants to the study. The study will also be widely promoted through extensive multicultural networks and ethnic media to ensure the sample is as representative as possible.

The sizeable samples, and the inclusion of international sites, will allow a unique set of comparisons to be made within a single study. This will provide a solid evidence-base for mapping social policies relating to youth with forced or voluntary migratory experiences, successful settlement, and psychological health and wellbeing in Australia, Canada and the USA. The proposed sample size will provide 80 percent power to determine differences of the sizes (i.e., effect sizes) listed in Table 1.

**Table 1:** Approximate differences (SD units) detectable with a 2-sided significance of 5% and power of 80% (given normally distributed measures), for selected comparisons of interest, based on an independent sample t-test.

Comparison of interest	N per group	Difference (unit of SD)
Between refugees and migrants	600	0.16
Between genders either within or between refugees and migrants	300	0.23
Between communities ignoring migration status	200	0.28
Between communities within or between refugees and migrants	100	0.38
Between genders within communities	100	0.38
Between Australia, the USA and Canada	600	0.16

This project is primarily exploratory, that is hypothesis generating rather than hypothesis testing. As such, sample size is only relevant with respect to the required accuracy of point estimates, and to allow for reasonably complex modelling without overfitting. The large sample sizes will in fact provide a minimum accuracy of +/- 4% with 95% confidence for any questionnaire item for youth with forced and voluntary migratory experiences separately, and +/- 3% for both groups combined. The sample sizes provided in Table 1 are therefore only indicative of potential comparisons that may be explored as part of the analysis.

#### Participant recruitment

**Fig 3:**
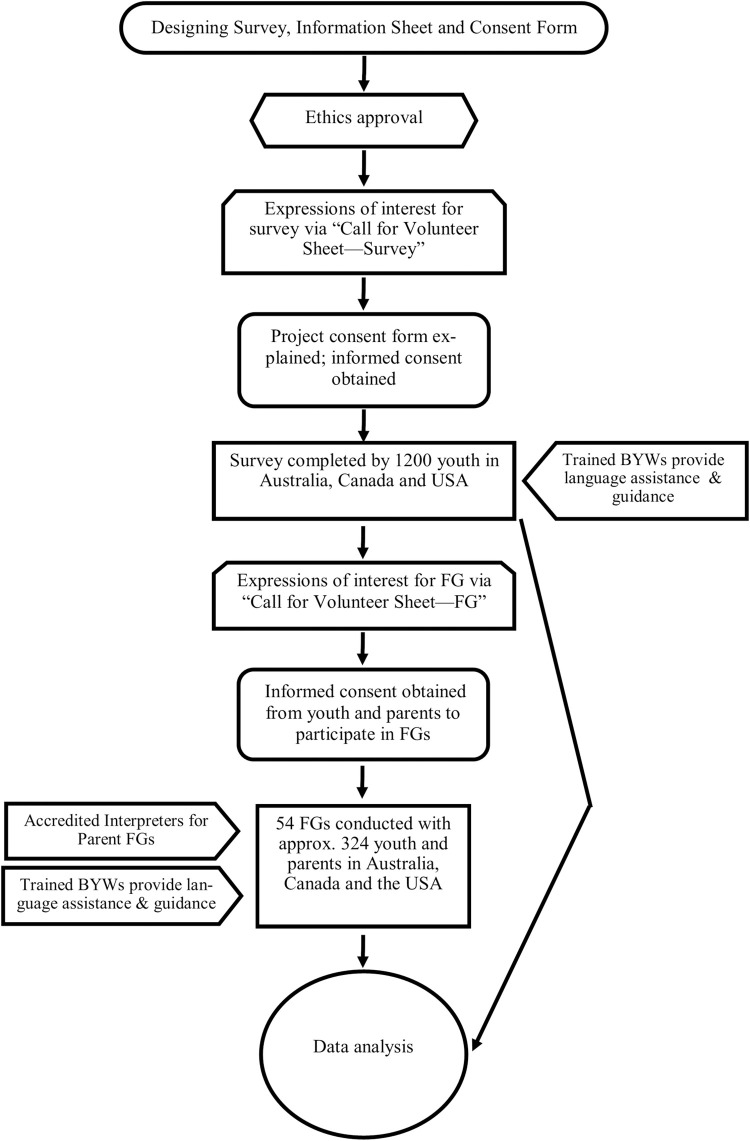
Study protocol.

Research team members will use a combination of strategies in multiple settings such as organizations, schools, community groups, and key persons who have extensive contact with settlement populations to assist with the recruitment of initial participants who in turn will be requested to refer potential participants to the study (Fig 3). Prior to recruitment, the research study will be widely promoted via project brochures, social gatherings and events organized by partner organizations, ethnic and mainstream media, and Bilingual Worker/Researchers (BW/Rs; described in next section). Research team members will also conduct community consultations with leaders of target communities to gain their support and trust for the research study. This strategy will be helpful in suggesting best practices to inform target communities of the study’s objectives.

Research team members will maintain a “Call for Volunteers Sheet—Survey” to record expressions of interest to participate in the survey study. This document will be translated into key target languages, if required. Youth and their parents/caregivers will be recruited for the FG study through a “Call for volunteers sheet—Focus Groups” presented to them after the completion of the survey questionnaire. For youth under 18 years, consent will be obtained from the youth as well as their parents/caregivers prior to participation. Parents/caregivers of youth will be recruited after obtaining youth consent for parents/caregivers’ participation in the FG discussions. However, youth without parents will not be excluded.

Participation in this research study will be entirely voluntary with participants informed of their right to withdraw at any point of time during the study, and this is included in the participant information sheet and consent form. At all times research team members will ensure that youth, their parents/caregivers, and key people in the target communities understand the study, and if they agree to be involved, that their privacy is protected at all times and their confidentiality is never violated. At all times, care will be taken to avoid the possibility of coercing people into participation.

#### Ethics and consent

As described in the relevant method sections herein, consent processes were followed with participants and with parents/caregivers as relevant for children under the age of 18. Consent and information will be provided in written format in 18 languages including English. Written consent will be sought and gained, with some scope for verbal consent when or if participants prefer this option, possibly due to literacy concerns. Ethics approval was granted from the University of South Australia Human Research Ethics Committee (approval ID: 203466) at the host university, and independent ethical procedures were followed with the partner organizations as per their own policy.

### Bilingual Workers/Researchers (BW/Rs)

As the survey will be administered in English, Bilingual Youth Workers (BW/Rs), one for each community represented, will be employed for the research project. The BW/Rs, who will speak the same language and belong to the same or similar ethnic background as the participants, will offer language assistance and support youth participants to complete the surveys. This method will enable enhanced communication so that nothing is missed in interpretation. Although the majority of BW/Rs will be recruited through partner organizations, suitable others with the requisite experience and from target communities, will also be recruited as and when required.

All BW/Rs will participate in an intensive training program which will be held a maximum of two weeks prior to commencing survey data collection. The training of BW/Rs will emphasize the importance of providing participants language assistance without directing participants about their answers. The training program will be repeated as and when the need arises either to small groups of newly recruited BW/Rs or individually. All BW/Rs will continue to receive refresher training at the request of BW/Rs s or when such a need is identified by the research team.

The researchers will prepare a comprehensive “Training Manual” for BW/Rs. The manual will provide background information on study, an explanation of the research questions and detailed instructions for guiding participants to complete the surveys including providing language assistance, and important ethical considerations. The training sessions will involve one-on-one coaching and skills development in conducting the survey cross-culturally, answering questions and queries, and dealing with difficult or adverse situations. The training manual will be given to each BW/R to guide them through their training and roles.

During the qualitative component of the study, BW/Rs will receive training to assist research team members to conduct FG discussions with both youth and their parents/caregivers as and when required. It is important to note, that the assistance afforded by BW/Rs will be with regard to language aspects and not with regards to survey or FG study content. BW/Rs will only be present to provide linguistic and cultural support for communication between the research team and participants, however, accredited interpreters will be engaged (and paid for by the study) if comprehensive interpreting is required.

### Data collection

#### Survey-youth 15–24 years

Data collection for the survey will be carried out at multiple project sites, in South Australia, Ontario, Canada, and California, USA. As much as possible, data will be collected according to the convenience and preference of participants. Data collection sites include university premises, partner organization and other agency premises, public libraries, and other public venues such as community centers. Each project site will have a research ground team (research coordinator, and BW/Rs) collaborating closely with the central research team based at the University of South Australia.

Youth who have expressed interest in participating in the study will be invited to one of several event/social gatherings organized at the premises of partner organizations. Agency premises were selected because we presumed that most participating youth will be familiar and feel comfortable at these premises. Food, music, and other fun activities will be organized at these events for participating youth. Several such events will be organized to facilitate survey data collection. Youth participating at these event/social gatherings will be invited to complete the survey together with a BW/R who speaks a language of their choice. This BW/R will guide the youth through completing survey, without compromising the confidentiality of participants’ responses; BW/Rs will be present only to explain words or concepts as relevant rather than to indicate how participants should answer.

Consent of the youth as well as a parent/caregiver of youth below 18 years will be obtained before survey data collection. Youth who do not wish to participate in an event/social gathering, will be contacted separately and alternate arrangements made for them to complete the survey with the assistance of a BW/R.

Participants will be given a choice to complete an online electronic version of the survey (i.e., Survey Monkey) or paper version of the survey, in English, but with language support from a BW/R. Youth participants will also be given the choice of completing the survey in English or in another language of choice. In the unlikely event, anyone selects a language other than English, their contact details will be recorded, and they will be invited to complete the survey with an accredited interpreter, at a later point of time.

The survey will be piloted with young people from the target communities in all three study locations. Feedback from the piloting will be incorporated into the final version of the survey. The final survey will take around 45 minutes to complete.

#### Focus groups: Youth and parents/cargivers

Each survey participant will be asked whether they would like to participate in an FG discussion of 60–90 minutes duration, at a later point of time. Their willingness for one parent/caregiver to be interviewed will also be ascertained. However, youth with no parents/caregivers will not be excluded from participation. The contact details of those who express interest as well as a willingness to have one of their parents/caregivers participate, will be entered in the “Call for volunteers sheet—Focus Groups”.

FG discussions will be conducted at partner organization premises, public libraries, or any other public place such as local council areas, convenient to participants. For participants below 18 years, the researchers will contact their parent/caregiver, afford information about the study, and independently obtain their consent for the participation of their child below 18 years.

Researchers will contact the parents/caregiver of youth 15–24 years who are identified by youth (Fig 2). After affording information about the study, and obtaining participant consent, the researchers will inform them of the date and time of the FG discussion they are allocated to. Only one parent/caregiver for each youth will be invited to participate.

Survey and FG participants will be offered a $20 gift voucher (in Australia) or an equivalent (in other locations) to thank them and recognize their time.

#### Translation of research material

While the information page and consent forms will be translated into target languages by an accredited interpreter from the official Translation and Interpretation Service (TIS), the survey questionnaires will be in English with BW/Rs providing language assistance required by participant youth. However, participants who request to complete the survey in a language other than English, will not be excluded from the study. As aforementioned, their details will be included in a “Survey Translation Sheet” and they will be invited to complete the survey with an accredited interpreter on a later date.

FG discussions with youth will be conducted in English with language assistance provided by BW/Rs. Since the majority of parents/caregivers are likely to be non-English-speakers, accredited interpreters will provide interpreting for FG discussions conducted with parents/caregivers. If required, the information and consent form relevant to parents/caregivers will also be audio recorded in the target languages for those parents/caregivers who do not have adequate language literacy in their native languages.

#### COVID-19 contingency plan

**Fig 4:**
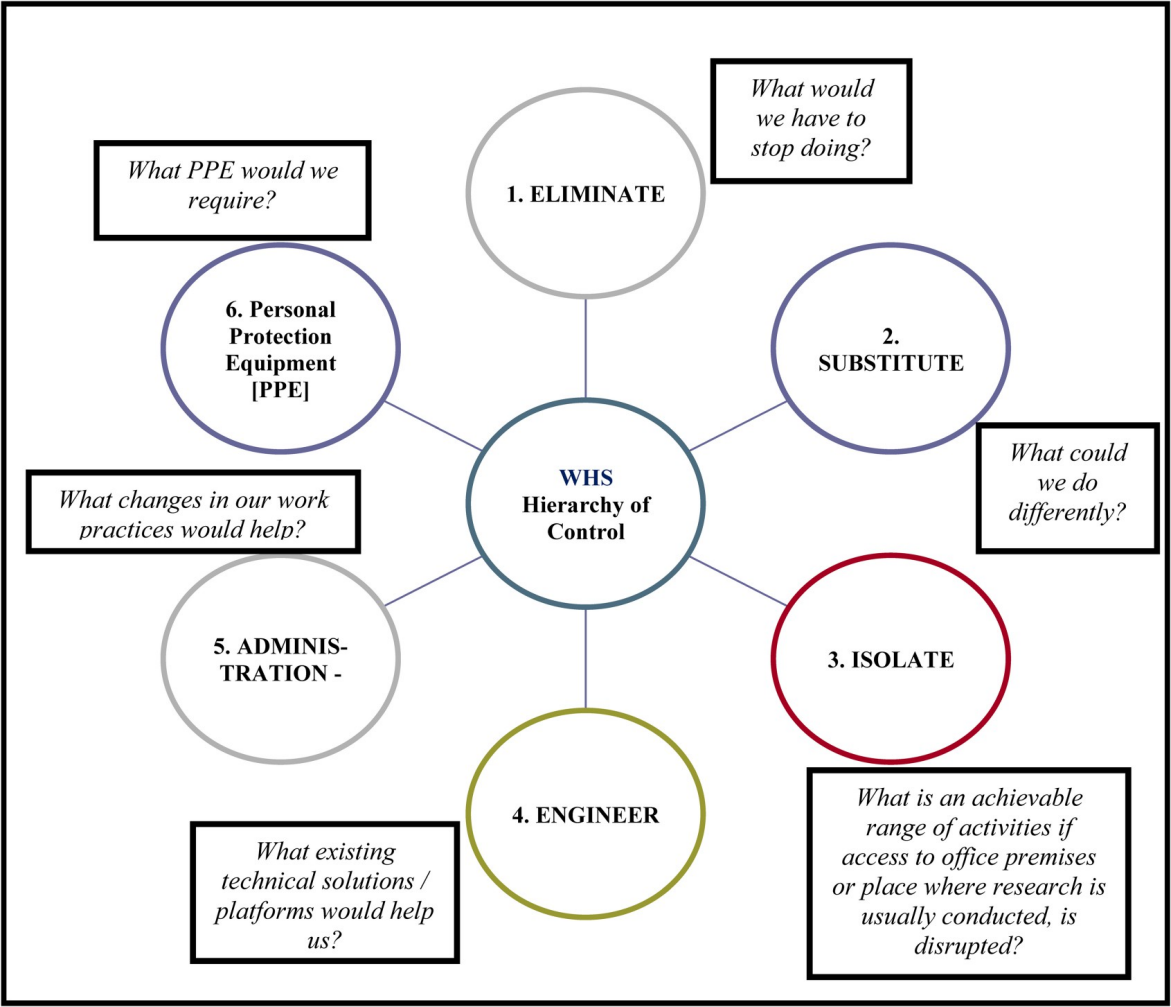
Work Health and Safety (WHS) Hierarchy of Control.

To ensure the safety of research team members, BW/Rs, and participants, and to mitigate the risk of COVID-19 transmission, we include a COVID-19 Contingency Plan for data collection based on the “Work Health and Safety (WHS) Hierarchy of Control” [70]. There are six elements of this WHS Hierarchy of Control to mitigate the risk of COVID-19. They are: Eliminate, Substitute, Isolate, Engineer, Administration, and the use of Personal Protective Equipment (PPE). Fig 4 is an illustration of the University’s WHS Hierarchy of Control.

Considering the vastly differing situations pertaining to COVID-19 among the three research sites we propose that Partner Investigators (PIs) at each site be responsible for selecting and activating the WHS Hierarchy of Controls as and when they deem such measures are required. We suggest that each partner organization adapt the COVID-19 Contingency Plan to optimally meet the needs of their unique situation at a particular point of time. We also suggest that they revise their selected WHS Hierarchy of Controls depending on the changes observed in their COVID-19 situation, with the passing of time. Hence, the Contingency Plan we propose is a guide only, in recognition that PIs and their research teams are the experts of their individual situations. PIs are encouraged to discuss the implementation of their respective WHS Hierarchy of Controls and plan of action with other members of the Project Management Committee including project lead (TZ) and research team member who is a leading Australian epidemiologist (AE).

At any point of time, before or during data collection, a PI could request a meeting of the Subcommittee Overseeing the COVID-19 Contingency Plan. They could do so, by informing the project lead or postdoc (TP) who will then organize a meeting of subcommittee members, to be held as soon as possible, on a date convenient to all/most. For an example of a Contingency Plan when face-to-face interaction is not possible please see Table 2.

**Table 2:** COVID-19 Contingency plan when face-to-face interaction is not possible.

***Project Promotion***	
**Pre-COVID-19**	**COVID-19 Contingency Plan**
Through project brochure, webpage, social gathering and events, and media. Through organizations, schools, community groups and key persons with extensive contact with the target populations. Community consultations attended by research team members. “Call for volunteers sheet”.	Widely distribute electronic copies of project brochure. Design state-of-the-art website to promote study among different stakeholders—settlement communities, service agencies, academics, and others. Promote project via social media. Discuss with partner agencies to promote study via agency websites as well as planned online activities such as conferences and training. Organize online discussions and Q&A sessions focusing on research project. Organize virtual consultations between research team and community leaders/elders. Maintaining electronic version of “Call for volunteers sheet” e.g., on project and partner agency websites.
***Training***	
**Pre-COVID-19**	**COVID-19 Contingency Plan**
BW/Rs representing target populations and recruited by partner organizations will participate in a group training program conducted face-to-face at partner organization premises. They will be afforded a hard copy of the “Training Manual”. Other BW/Rs recruited by research team will be provided with individual, face-to-face training.	Organize with partner organizations to conduct virtual training of BW/Rs via Zoom. Training will be conducted for each agency separately or alternatively, all BW/Rs could be trained via one large virtual training session. Follow-up refresher training via Zoom to be provided to smaller groups of BW/Rs, as and when required. Provide electronic version of “Training Manual” to all BW/Rs/relevant agency staff.
***Data Collection***	
**Pre-COVID-19**	**COVID-19 Contingency Plan**
At agency premises, youth to participate in event/social gathering and complete surveys guided by a BW/R who provides required language assistance. Survey data from other youth to be collected by BW/Rs, face-to-face, at multiple venues including university, agency premises, public libraries other public venues. Participants to be given choice of completing electronic or paper version of survey.	Partner agencies organize to collect survey data via Zoom. BW/Rs could either guide participants to complete electronic version of survey or record participants’ responses on paper surveys. At conclusion of survey, BW/Rs recording participants’ consent to participate in FG discussion including securely recording contact details provided by participants.

If PIs in consultation with the Project Management Committee decide that face-to-face contact can be safely carried out and there are no official regulations restricting such contact, research may continue utilizing a combination of the original research methods and the COVID-19 Contingency Plan. PIs and their research teams will ensure at all time that they comply with regulations pertaining to such contact in place, in their respective jurisdictions. To this end, partner agencies will also follow their own COVID-19 safety plans.

### Survey instruments and measures

The survey study includes seven sections, A to G. All youth participants aged 15–24 years will be requested to complete the seven sections, although some sections may not be relevant to all youth (e.g., the ‘employment’ section will not be relevant to youth not employed). The survey will be completed by youth participants, in the presence of trained BW/Rs who will support youth to respond to survey items including offering necessary language assistance.

The instruments selected for inclusion in the survey (except the question on the impact of COVID-19) have previously been extensively used in research studies conducted in several countries and in diverse cultural contexts, sometimes after necessary adaptation, and in many cases have been translated into and conducted using numerous languages (e.g. MIRIPS, measuring acculturation and adaptation, CD-RISC and SDQ, measuring resilience and strengths and difficulties respectively). While reliability and validity data are available for each of these instruments, for many cultural and country contexts, it has not been possible to find such data for all the linguistic and cultural groups anticipated to participate in this study. In the absence of such data, we have selected instruments that have been widely used in cross cultural research. The final version of the complete survey as utilized in this study will be decided in consultation with the project partners and PIs, using an iterative process after piloting with youth participants in each project location.

The seven survey sections are as follows:

PART A—Sociodemographic details titled: “About You”

PART B—Acculturation and Adaptation: “Life in Australia” (or Canada/the USA)”

PART C—Emotional Health and Wellbeing: “Your Experiences, Thoughts and Feelings”.

PART D—Family Functioning: “Your Family”

PART E—Educational experiences: “Your Educational Experiences”

PART F—Service Utilization: “Do You Ask for Help?”

PART G—Employment Experiences: “Your Employment Experiences”

#### Sociodemographic details

Information about young people’s demographic characteristics (age, gender, cultural/ethnic background, family structure, experience in refugee camp or immigration detention center, etc.) will be obtained by adapting a questionnaire developed for this purpose in the Research and Evaluation Unit, Women’s and Children and Hospital (South Australia) and used extensively in previous studies with youth.

#### Acculturation and adaptation

Acculturation and adaptation within Australian (or Canadian or American) society will be assessed by several questionnaires included in the Mutual Intercultural Relations in Plural Societies (MIRIPS) study and adapted for purposes of this study. Initiated by Professor John W. Berry, the MIRIPS study was a large-scale study focusing on intercultural relations within complex, culturally plural, societies. The study has been conducted across 26 countries including Australia, and the scales have hence been substantially tested for reliability and validity across cultural settings [71]. Sections of the MIRIPS questionnaire relevant and included in this study are 14 items assessing cultural identity, 16 items assessing acculturation, 10 items assessing self-esteem, five items assessing satisfaction with life, and seven items assessing perceived discrimination. All sections prompt a response with a Likert scale.

#### Emotional health and wellbeing

Two measures of emotional health and wellbeing of youth will be investigated in the survey. They are (i) resilience and (ii) strengths and difficulties. Resilience will be measured using the 10-item abridged version of the Conner-Davidson Resilience Scale [72]. Strengths and difficulties will be measured by the 25-item Strengths and Difficulties Questionnaire [73]. Both instruments have excellent psychometric properties, have been widely used, and have been translated into numerous languages to be used in varying cultural contexts.

#### Family functioning

Youth participants’ family functioning will be assessed using the General Functioning Scale of the McMaster Family Assessment Device (FAD) [74]. The complete McMaster FAD instrument has excellent psychometric properties, and wide use, including being translated into 14 languages. Because of its length (53 items) only 1 scale—the 12-item General Functioning Scale—from the FAD was included in this study. This Scale has been recommended for use as a standalone and brief measure of overall family functioning with excellent psychometric properties [75,76].

#### Schooling experiences

Youth’ educational experiences will be assessed utilizing several items from the National Schools Opinion Survey (Australia), assessing school satisfaction and the Life in School Checklist assessing bullying at school [77,78], with six Likert response items each assessing experiences at school and bullying, alongside two additional items on future educational and occupational/career plans.

#### Service utilization

There are few high-quality questionnaires available to assess formal (official health services) and informal (community, social and religious) help-seeking among youth. The research study adopted several items in the child and adolescent national survey [79], to assess informal and formal help seeking behavior among youth, 15–24 years. The help-seeking section is designed to answer three main questions: (1) What services/professionals youth access for emotional and behavioral issues and problems? (2) What type of help was provided through these services? And (3) What barriers prevent youth accessing necessary services?

#### Employment experiences

The engagement of youth in some form of employment will be ascertained using relevant sections of the Australian Workplace Barometer (AWB) [80] developed by Dollard and colleagues of the Centre for Workplace Excellence at the University of South Australia. Sections of the AWB included in the survey are: Nature of employment, physical and emotional job demands, as well as organizational bullying and harassment. There are 11 questions in total drawn from the AWB in this section, including four which include multiple items within a Likert scale.

#### Impact of COVID-19

In addition, every Section except the sociodemographic Section A, includes four items exploring the impact of the COVID-19 pandemic on participants’ experiences in each area (i.e., acculturation and adaptation, emotional health and wellbeing, family functioning, education experiences, service utilization, and employment experiences). An example of an item is: “please indicate on the following scale, how much COVID-19 affected your employment experiences 1.Not at all / 2.Little / 3.Somewhat / 4.Quite a bit / 5.Very much”.

#### Focus group instruments and measures

FG questions will be based on the areas of inquiry of the survey. FGs will be based on a semi-structured protocol with questions and prompts to be finalized after the completion of survey.

### Ethical considerations

#### Ethics approvals

The Ethics Protocol of the research study had already been approved by the University of South Australia’s Human Research Ethics Committee as adhering to the requirements of the National Statement on Ethical Conduct in Human Research in Australia. In compliance, members of the Project Management Committee will ensure that all aspects of the research project will be performed in accordance with the ethical principles laid down in the aforementioned National Statement and University guidelines on conducting research. Members of the Project Management Committee will also be responsible for informing the University’s Ethics Committee of (a) any amendments to the approved project protocol and (b) of any serious adverse events arising when conducting the research project by contacting the Executive Officer of the University of South Australia’s Ethics Committee. Ethics clearance has also been obtained at the two international sites. In the USA, the partner organization had already been endorsed by the U.S. Department of Health and Human Services to conduct IRB project reviews. Accordingly, the research project was approved by the agency’s IRB committee after necessary amendments to the research protocol. All U.S. research team members also received certification from the U.S. Department of Health and Human Services for Human Research Protection Training. In Canada, the research protocol was endorsed by the partner organization’s Board of Directors subsequent to approval by the University’s Ethics Committee. In addition, considering the sensitive nature of the overall study, special effort will be taken to avoid any situations and contexts of power imbalance when conducting surveys (i.e. not to conduct research in the context of dual relationships or providing compensation that could be viewed as coercion).

#### Participant consent process

Youth and parent or caregiver participants in this study can consent to participation independently if they are over the age of 18 years in any of the data collection countries. Youth under the age of 18 will be asked to assent to participation, however official consent will be sought from a parent or caregiver. Participants (and parent/caregivers for youth under 18) will be provided with written copies of the information sheets in the first instance. They will additionally be provided with a verbal explanation of the information and afforded time to consider the study and to ask any questions if they so choose. The information and consent forms have been translated into target languages for participants or parent/caregivers who are literate in languages other than English, and in addition certified interpreters will be provided by the study team should any language needs not be met through these translated copies. If the participant and/or parent/caregiver consents to participation, a signature will be requested and this form will additionally be sighted and signed by a member of the research team or BW/Rs. Provision for verbal consent is noted on the consent forms, as some participants may need to give consent verbally and these forms will be signed by a researcher or BW/R.

## Results

### Quantitative analysis

Immediately after the conclusion of data collection, exploratory data analysis will be used to explore and summarize the main characteristics of the dataset. For this purpose, statistical graphs, and other data visualization methods will be used. This initial exploratory analysis will be used to generate hypotheses and models in response to the research questions. Thereafter initial data analysis will be conducted involving checking assumptions required for model fitting, checking for measurement invariance (across the 3 project sites and 4 migration regions), hypothesis testing, handling missing values and making transformations of variables, if required. Table 3 contains the research questions that will be used to formulate hypotheses, together with the methods and statistical models likely to be used.

**Table 3:** Research questions, methods, and statistical models.

	Research question	Methods	Statistical models
1.	What are the settlement experiences^i^ of youth residing in Australia and how do these experiences compare with the settlement experiences of youth in Canada and USA?	Survey	cluster analysis, multiple regression, analysis of variance and covariance
2.	What are the relationships between key demographic factors^ii^ acculturation and adaptation, mental health, family functioning, education and employment experiences of youth in Australia, Canada and USA?	Survey	Multiple regression with mediation and moderation analysis, path analysis using structural equation modelling
3.	What are the contextual, structural and cultural factors identified by youth and their parents/caregivers as influencing their settlement and wellbeing?	FG / Survey	Qualitative Thematic analysis
4.	What are the experiences of youth and their families accessing and utilizing services and how do they navigate around the barriers (if any) to service access and utilization?	FG / Survey	Qualitative Thematic analysis
5.	What are the recommendations for improved access and utilization of services?	FG	Qualitative Thematic analysis

^i^ Acculturation and adaptation, mental health, family functioning, education and employment experiences and service utilization.

^I^ Gender, Age, time in settlement country, migration pathway (forced and volutary), migration region (Asia, Africa, Middle East etc.).

An initial analysis will contrast youth with forced and voluntary migratory experiences in Australia, Canada, and the USA. Descriptive analysis will be conducted for major constructs before using inferential statistics, such as cluster analysis, multiple regression, and analysis of variance. Further analyses will be undertaken separately within the two immigrant groups as well as, the three project sites Australia, Canada, and the USA to determine risk factors for poor mental health and psychosocial adjustment.

Standard statistical techniques will be used to identify the prevalence of psychosocial problems reported by youth, including categorical approaches (e.g., proportion of youth scoring above recommended cut-off scores on checklists) and the use of continuous scores (e.g., the mean scores on relevant questionnaire scales).

Time since arrival in Australia, Canada and the USA will be used as a covariate in analyses where appropriate. Age and gender will be treated as a continuous covariate and as a categorical variable, the latter allowing the investigation of ‘interpretable’ measures of interaction. An initial comparison will be made examining the distributions of scores for all groups using both graphical techniques (e.g., cumulative distribution plots) and statistical tests of significance on either transformed scores or using generalized linear modelling which acknowledges that the distribution of these scores is not necessarily Normal (Gaussian).

Structural equation modelling will be conducted to examine the pattern of relationships among key variables. However, several other factors, such as family functioning, psychological adjustment, perceived discrimination, and education and employment experiences are also important determinants of psychosocial problems, and any differences between the groups will be explored using regression modelling. All point estimates will include confidence intervals, and effect sizes will be provided for main comparisons. The statistical packages SPSS and Stata will be used for analyses.

### Qualitative analysis

Qualitative FG group data will be analyzed thematically following the principles and procedures suggested by Krueger and Casey [81] and also Braun and Clarke [82–84]. The researchers acknowledge limitations to thematic analysis in terms of the positioning of themselves and due to their own experiences and understandings. The research team will work in collaboration, drawing on the diverse disciplinary and experiential backgrounds of the large team. The team has a range of migration backgrounds and cultural and linguistic viewpoints from which to draw on during analysis and will practice reflexivity throughout the process. Themes will be inductively identified and discussed collectively amongst the research team, through initial reading of transcripts and listening to recordings, to iteratively defining codes and coding the data using software [84].

The FG discussions will be audio taped and the English portions transcribed verbatim. As the project will engage accredited interpreters for the sessions conducted in a language other than English, we can be assured that the interpretation is of the highest quality, however as the languages spoken by the research team are limited to five languages only, we will not have facility to cross-check the interpreting and the English portion of the recordings will be relied upon for data analysis.

The data will be presented in the form of detailed description, using case illustrations, paraphrases, and direct quotations from the themes identified while maintaining anonymity of the participants. The qualitative data management and analysis program NVivo 12 will be used to code and to facilitate analysis of a large amount of textual data.

### Mixed methods analysis

Synthesis of the mixed-methods data will be achieved through integration of the quantitative survey data from youth and the qualitative FG data from youth and parents/caregivers. It is anticipated that the quantitative data analysis will enable a broad understanding of key trends and relationships between study variables. The FG data analysis will subsequently offer opportunity for in-depth examination of trends that were observed from the survey, as well as offering new insights into key topics of interest to participants. Synthesis of these two data sets will provide a detailed understanding of what was important to participants and why [69]. This mixed-methods approach has been noted as effective for use with diverse participant cohorts, such as is the case for this study, and it aligns with the transformative-advocacy methodology as it allows for researchers to understand complex contexts and experiences through interpreting similarly complex and rich quantitative and qualitative data [85].

## Discussion

The COVID-19 pandemic had led to a major pause of humanitarian and other settlement programs in settlement countries such as Australia and a resultant reduction in the intake of migrants into these countries. This is despite the ever increasing number of people being displaced within their own borders as well as across national borders around the world [86]. However, the future population growth and economic productivity of countries such as Australia, Canada, and the USA will depend on the successful settlement and wellbeing of these settlement populations. While the Australian Federal Government’s Longitudinal Survey of Immigrants to Australia, and the new Longitudinal Survey of Humanitarian Migrants (2013–2018) attests to the importance of understanding their settlement trajectories, such large-scale surveys focus on the adult population while our study focuses on youth, thereby providing new information to support the development of evidence-based policy and service interventions. Studies of youth are scarce even though it is widely known that migration and its accompanying stressors can have long-term effects on settlement, psychological health, and wellbeing.

Most youth studies are small in scale, restricted to a single cultural group; focus on either people who immigrate by choice or are forced to immigrate; the voice of adults; and a single area of investigation; and many are atheoretical. This research will address these limitations by collecting data about a large number of youth drawn from several ethnic communities, including both people who immigrate by choice or are forced to do so, covering four major areas of investigation, and grounding the investigation in theory. It includes sizeable samples from each population group, providing a rare opportunity for making comparisons in a single study. The support needs of youth differ widely according to pre- and post-migration context and other factors [60]. Understanding the nature and extent of these differences is important for policy, service provision and the extension of theory.

The research will focus exclusively on youth, a group that is often overlooked in the dichotomy between children and adults, and one that requires evidence-informed support. Recent studies have found they may be at increased risk of a range of other problems associated with migration and resettlement e.g., family problems, intergenerational conflict, adjustment problems, homelessness, truancy, offending, violence, drug and alcohol misuse and unsafe sexual practices and pregnancies [50]. However, there is a paucity of research that provides evidence-based recommendations for policy and service-provision, a limitation that this research will address.

A unique feature of this study is that it will be conducted in three countries: Australia (South Australia), Canada (Ontario) and the USA (California), allowing for an international comparison and in-depth understanding of the settlement experiences and outcomes of youth as well as their patterns of service utilization. South Australia provides a broadly representative sample of youth with forced migratory experiences since it takes a significant share of humanitarian settlers [62]. Prior to the COVID-19 pandemic, Canada was expecting one million new migrants between 2018–2020 under its Multi-Year Levels Immigration Plan. In 2018 the Province of Ontario had the highest number of migrants compared to other Canadian provinces A multi-country focus will allow us to investigate local, national, and international trends, with the findings being of direct relevance to all countries.

This study has the potential to significantly advance the field of research. The study design allows a number of important phenomena (e.g., the immigrant paradox, different migration pathways) and key questions (e.g., the relationship between settlement outcomes and wellbeing; the relationship between the context of migration and settlement and wellbeing; the relationship between positive and negative domains of wellbeing) to be addressed, and the generalizability of the findings across different communities and countries to be tested. This has not been done in any other single study.

## Conclusion

This research study is expected to help bridge notable gaps between research, policy, and practice pertaining to the settlement experiences and wellbeing of young people. While the growing cultural diversity in countries such as Australia, Canada, and the USA has given rise to a range of policies and plans that recognize the importance of promotion, prevention, and early intervention for mental health for young people settling into these countries, policy and service developments have largely occurred in a knowledge vacuum. Research continues to trail behind policy and practice, particularly in relation to youth settlement. We aim to reverse that trend through providing evidence-based applications to improve the psychological health and wellbeing of young people and their families. The goal of this research project is to explore new trends, identify issues globally, nationally, and locally, as well as inconsistencies with policies and practices. The research findings will be used to inform policy design around support for young people–including health, education, employment, and social/community support policies tailored to the specific needs of young people with forced and voluntary migration experiences–including “bottom-up” policy insights for local policymakers and community groups as well as more traditional “top-down” national and state policy. It will also provide evidence to support a comparative international analysis of policies to support young people settling into Australia, Canada, and the USA.
